# Determinants of ureteral obstruction after percutaneous nephrolithotomy

**DOI:** 10.1007/s00240-022-01365-8

**Published:** 2022-10-14

**Authors:** Harry H. Lee, Heiko Yang, Patrick Martin-Tuite, Rei Unno, Fadl Hamouche, Justin Ahn, David Bayne, Marshall Stoller, Thomas Chi

**Affiliations:** 1 Department of Urology, University of California, San Francisco, 400 Parnassus Ave, A632, San Francisco, CA 94143-0738 USA; 2 Department of Urology, New York University Langone Health, New York, NY USA; 3 School of Medicine, University of California, San Francisco, San Francisco, CA USA

**Keywords:** Kidney stones, Percutaneous nephrolithotomy, Ureteral obstruction, Totally tubeless

## Abstract

**Background:**

Ureteral obstruction after percutaneous nephrolithotomy (PCNL) may require prolonged drainage with a nephrostomy tube (NT) or ureteral stent, but it is not well understood how and why this occurs. The goal of this study was to identify risk factors associated with postoperative ureteral obstruction to help guide drainage tube selection.

**Methods:**

Prospective data from adult patients enrolled in the Registry for Stones of the Kidney and Ureter (ReSKU) who underwent PCNL from 2016 to 2020 were used. Patients who had postoperative NTs with antegrade imaging-based flow assessment on postoperative day one (POD1) were included. Patients with transplanted kidneys or those without appropriate preoperative imaging were excluded. We assessed the association between patient demographics, stone characteristics, and intraoperative factors using POD1 antegrade flow, a proxy for ureteral patency, as the primary outcome. Stepwise selection was used to develop a multivariate logistic regression model controlling for BMI, stone location, stone burden, ipsilateral ureteroscopy (URS), access location, estimated blood loss, and operative time.

**Results:**

We analyzed 241 cases for this study; 204 (84.6%) had a visual clearance of stone. Antegrade flow on POD1 was absent in 76 cases (31.5%). A multivariate logistic regression model found that stones located anywhere other than in the renal pelvis (OR 2.63, 95% CI 1.29–5.53; *p* = 0.01), non-lower pole access (OR 2.81, 95% CI 1.42–5.74; *p* < 0.01), and concurrent ipsilateral URS (OR 2.17, 95% CI 1.02–4.65; *p* = 0.05) increased the likelihood of obstruction. BMI, pre-operative stone burden, EBL, and operative time did not affect antegrade flow outcomes.

**Conclusion:**

Concurrent ipsilateral URS, absence of stones in the renal pelvis, and non-lower pole access are associated with increased likelihood of ureteral obstruction after PCNL. Access location appears to be the strongest predictor. Recognizing these risk factors can be helpful in guiding postoperative tube management.

**Supplementary Information:**

The online version contains supplementary material available at 10.1007/s00240-022-01365-8.

## Introduction

Alleviating transient postoperative ureteral obstruction is one of the primary indications for nephrostomy tube (NT) or ureteral stent placement after percutaneous nephrolithotomy (PCNL). Although PCNL can be performed without leaving a drainage tube, evidence to support this practice is based on studies using narrow patient selection criteria [1–4]. Until more is understood about the mechanism of postoperative ureteral obstruction and how it might be predicted or avoided, the practice of routinely leaving drainage tubes remains the standard of care [5, 6].

It has been postulated that intraoperative ureteral manipulation causes tissue edema, particularly at the ureteropelvic junction (UPJ), and is the main driver of transient postoperative ureteral obstruction [7]. As there is little data to confirm this hypothesis, this is a promising area for further investigation. Since tissue trauma and edema are challenging parameters to quantify, we designed this study to examine whether objective variables such as stone size, location, choice of access, and concomitant ipsilateral ureteroscopy might be associated with postoperative obstruction as assessed with a routine antegrade flow study. These variables correlate with the degree of intraoperative tissue manipulation [7–9]. Our aim was to identify relevant risk factors for ureteral obstruction to improve patient selection for tubeless PCNL. We hypothesized that the risk of developing postoperative ureteral obstruction increases with greater stone burden, higher stone complexity, and more instrumentation of the UPJ and ureter.

## Methods

### Study design

Prospective data was analyzed from adult patients enrolled in the Registry for Stones of the Kidney and Ureter (ReSKU) who underwent PCNL using pneumatic and ultrasonic lithotripsy from 2016 to 2020. Patients who had postoperative nephrostomy tubes and underwent antegrade imaging-based flow assessment on a postoperative day one (POD1) were included. Patients without preoperative imaging within 6 months of the procedure or who had transplanted kidneys were excluded. Antegrade flow from the kidney to the bladder as determined by fluoroscopic antegrade nephrostogram (ANG), contrast-enhanced ultrasound (CEUS), or methylene blue dye test was analyzed as the primary outcome. Ureteral obstruction was defined as lack of antegrade flow on any of these tests. The modality utilized was based on provider preference. These techniques have been described in previous studies and have been found to have comparable accuracy in detecting ureteral obstruction [10–12]. Representative images for antegrade nephrostogram and CEUS are shown in Supplemental Figure 1. Within the context of post-operative antegrade flow assessment, no patients analyzed in this study had double-J stents following PCNL.

Patient demographics, preoperative stone characteristics, and intraoperative characteristics were included in the analysis. Each patient’s stone burden was calculated as the sum of the longest diameters of individual stones in the affected kidney. Preoperative hydronephrosis was ascertained from the radiology report and confirmed with manual review. Stone location was scored based on a manual review of imaging by two urologists; representative images are shown in Supplemental Figure 2. Stones were categorized as “upper pole calyx,” “mid pole calyx,” “lower pole calyx,” “renal pelvis,” or “ureter” as best described. Multiple stones in the same general location were scored as a single location while stones in different locations were scored as separate locations. Tract number, diameter, and location (upper, mid, or lower kidney), presence of preoperative ureteral stent, concurrent ipsilateral antegrade and/or retrograde ureteroscopy, stone clearance, and estimated blood loss (EBL) were extracted from the operative note. For stone clearance, both “visually stone free” and “fragments small enough to pass” identified on flexible nephroscopy were considered to be “stone free” while any mention of a larger stone fragment or stone requiring subsequent intervention was considered to be “not stone free.” With the understanding that surgeon assessment of stone-free status is not reliable, the metric was primarily used to distinguish whether there was a significant stone burden at the end of surgery that could influence tube management or affect patency rates. A correlation matrix of all variables is shown in Supplemental Figure 3.

### Statistical analysis

R (Version 1.4.1103) was used for all statistical analyses. Chi-squared tests and Student *t* tests were used as appropriate to conduct univariate analyses of patient demographics, preoperative variables, intraoperative variables. Stepwise selection was used to develop a multivariate logistic regression model controlling for BMI, stone location, stone burden, ipsilateral ureteroscopy (URS), access location, estimated blood loss, and operative time. A *p*-value < 0.05 was deemed significant.

## Results

**Fig. 1:**
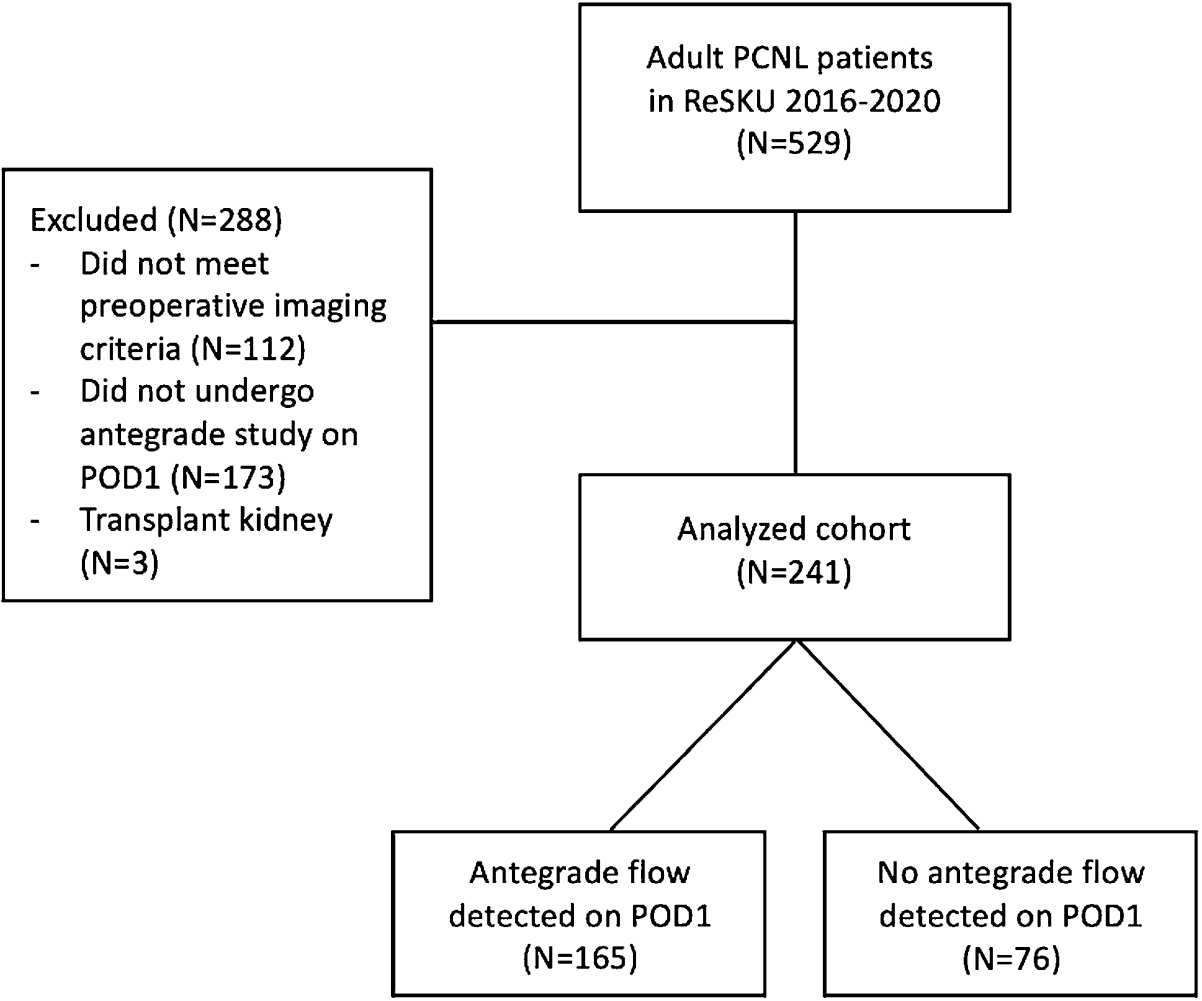
Study flowchart. *PCNL* percutaneous nephrolithotomy, *ReSKU* registry for stones of the kidney and ureter, *POD1* postoperative day 1

We identified 241 PCNL cases in 215 unique patients in the ReSKU database that met all study criteria (Fig. 1). Tract sizes ranged from 10 to 30 Fr, with a median of 24 Fr. Stone clearance was documented in 204 cases (84.6%). Antegrade flow on POD1 was detected in 165 cases (68.5%) while 76 (31.5%) had an obstruction (Table 1). ANGs were the most frequently used modality to assess ureteral patency (41.9%), followed by CEUS (35.7%) and the methylene blue dye test (22.4%) (Table 2). Of the 165 cases with the antegrade flow on POD1, 134 patients (81.2%) had their NTs removed on the same day without complication, 7 patients (4.2%) exhibited symptoms suggestive of obstruction following NT removal (e.g., flank pain and fever), and 24 patients (14.5%) kept their NTs for other reasons.

**Table 1:** Patient characteristics

	Passed antegrade (%)/(SD)	Failed antegrade (%)/(SD)
*N*	165	76
Demographics		
Age (years)	53.8 (16.8)	56.4 (14.6)
Sex		
Male	70 (42.4%)	40 (52.6%)
Female	95 (57.6%)	36 (47.4%)
Other	0 (0%)	0 (0%)
Race		
Asian or Pacific Islander	24 (14.5%)	12 (15.8%)
Black	11 (6.7%)	7 (9.2%)
Hispanic	22 (13.3%)	7 (9.2%)
White	94 (57%)	41 (53.9%)
Other	13 (7.9%)	9 (11.8%)
BMI	29.4 (7.2)	31.1 (9.2)
ASA		
I	13 (7.9%)	5 (6.6%)
II	106 (64.2%)	45 (59.2%)
III	45 (27.3)	26 (34.2%)
IV	1 (0.6%)	0 (0%)

**Table 2:** Study modality used to determine antegrade flow

	Passed antegrade (%)	Failed antegrade (%)	Total (%)
*N*	165	76	241
Fluoroscopic antegrade nephrostogram	65 (39.4%)	36 (47.4%)	101 (41.9%)
Contrast-enhanced ultrasound (CEUS)	58 (35.2%)	28 (36.8%)	86 (35.7%)
Methylene blue dye test	42 (25.5%)	12 (15.8%)	54 (22.4%)

Univariate analysis of stone characteristics and intraoperative variables revealed that mid and upper kidney access (*p* = 0.03) and concurrent ipsilateral URS (*p* = 0.04) were associated with an increased risk of obstruction (Table 3). We did not observe any significant associations between patient demographics (e.g., age, sex, race, BMI, ASA) and obstruction. Using a multivariate logistic regression model, we found that mid or upper kidney access (OR 2.81, 95% CI 1.42–5.74; *p* < 0.01), ipsilateral URS (OR 2.17, 95% CI 1.02–4.65; *p* = 0.05), and stones located anywhere other than in the renal pelvis (OR 2.63, 95% CI 1.29–5.53; *p* = 0.01), were associated with an increased likelihood of obstruction, while BMI, pre-operative stone burden, EBL, and operative time were not significantly associated (Table 4).

**Table 3:** Stone characteristics and intraoperative variables

	Passed antegrade (%)/(SD)	Failed antegrade (%)/(SD)	*p* value
*N*	165	76	
Pre-operative variables			
Laterality of stone			0.85
Unilateral	153 (92.7%)	71 (93.4%)	
Bilateral	12 (7.3%)	5 (6.6%)	
Pre-operative hydronephrosis	96 (58.2%)	44 (57.9%)	0.97
Stone location			0.14
Upper pole	30 (18.2%)	12 (15.8%)	
Middle pole	27 (10.2%)	21 (27.6%)	
Lower pole	83 (50.3%)	48 (63.2%)	
Renal pelvis	99 (60%)	34 (44.7%)	
Ureter	29 (10.9%)	14 (18.4%)	
Total pre-operative stone burden (cm)	3.2 (2.1)	3.6 (2.9)	0.34
Pre-operative ureteral stent	18 (10.9%)	4 (5.3%)	0.16
Intra-operative variables			
Ipsilateral URS	34 (20.6%)	26 (34.2%)	0.035
PCNL tract			
Tract size			0.3
10	5 (3.2%)	2 (2.7%)	
12	0 (0%)	1 (1.3%)	
14	1 (0.6%)	2 (2.7%)	
16	6 (3.8%)	0 (0%)	
24	134 (84.8%)	64 (85.3%)	
28	1 (0.6%)	0 (0%)	
30	11 (7.0%)	6 (7.1%)	
Number of tracts			0.86
1	139 (84.2%)	66 (86.8%)	
2	23 (13.9%)	9 (11.8%)	
≥ 3	3 (1.8%)	1 (1.3%)	
Tract location			0.034
Upper pole	42 (25.5%)	28 (36.8%)	
Middle pole	52 (31.5%)	28 (36.8%)	
Lower pole	93 (56.4%)	28 (36.8%)	
Visualization of stone clearance	141 (85.5%)	63 (82.9%)	0.22
EBL	64.9 (67)	51.7 (51.8)	0.1
Operative time (mins)	110.3 (38.9)	122 (46.7)	0.091

**Table 4:** Multivariate analysis

	OR	CI: 2.5%	CI: 97.5%	*p*-value
BMI	1.04	1.00	1.09	0.07
Pre-operative stone burden (cm)	1.14	0.99	1.32	0.08
Ipsilateral URS	2.17	1.02	4.65	0.045
Tract location—non-lower pole	2.81	1.42	5.74	0.004
EBL	1.00	0.99	1.00	0.18
Operative time	1.01	1.00	1.02	0.09
Stone location—non-renal pelvis	2.63	1.29	5.53	0.009

## Discussion

In this study, we demonstrate that stone location, selection of access during PCNL, and concurrent ipsilateral ureteroscopy are associated with postoperative obstruction after PCNL in a multivariate model. In addition to shedding light on a potential mechanism for how postoperative ureteral obstruction develops, these results suggest that ureteral obstruction necessitating postoperative drainage tubes might be predictable (Table 5).

**Table 5:** Summary of variables tested and their association to the risk of obstruction on POD1 of PCNL

Increased risk of obstruction	No observed association	Decreased risk of obstruction
Stone location not in renal pelvis	Age	Stone location in renal pelvis
Upper or mid kidney access	Sex	Lower kidney access
Ipsilateral ureteroscopy	BMI	
	ASA	
	Stone laterality	
	Pre-operative hydronephrosis	
	Stones in multiple location	
	Number of tracts	
	Ipsilateral URS	
	Visual clearance of stone	
	Operative time	
	EBL Higher stone burden Pre-operative stent	

Our data support the hypothesis that the UPJ and ureter contribute to ureteral obstruction. Renal access from the mid and upper kidney facilitate antegrade access to the UPJ and ureter, thereby increasing the likelihood of mechanical trauma to the area during lithotripsy. Concurrent ureteroscopy by definition results in more ureteral instrumentation. Of note, factors such as stone burden and operative time, although not found to be statistically significant, trend towards an association with postoperative obstruction with a *p*-value of 0.08 and 0.09, respectively. One would expect with greater stone burden and operative time, ureteral instrumentation and consequent tissue trauma would also increase.

It is counterintuitive to note that the absence of stones in the renal pelvis would be associated with obstruction. One could argue that stones in the renal pelvis would lead to more trauma in the pelvis and UPJ. An alternative hypothesis, however, is that pre-existing irritation of the UPJ from a renal pelvis stone mitigates the degree of new edema caused by instrumentation. This explanation would also account for why preoperative hydronephrosis associated with ureteral stones was also not found to be correlated with postoperative obstruction.

The interpretation of these data should be tempered by the following limitations: first, antegrade studies performed on POD1 do not provide insight into the immediate postoperative state of the ureter, and we are likely underestimating the number of patients who develop transient postoperative ureteral obstruction. It is also worth mentioning that the binary nature of the antegrade exam does not fully capture the complexity of ureteral patency. For instance, time of transit is typically not documented and may be a determining factor for the small percentage of patients who develop obstructive symptoms even after a contrast agent or dye is detected in the bladder.

Another major limitation is that we lack an objective measure of UPJ or ureteral manipulation to fully support the tissue trauma hypothesis of ureteral obstruction. While many of the variables used in our study act as surrogates, they fall short of quantifying actual tissue trauma. Within this context, our study was further limited as we did not assess for stone hardness, duration of preoperative ureteral obstruction, and different energy modalities used (i.e., holmium laser, pneumatic, or ultrasonic lithotripsies), all variables that may influence ureteral patency. A prospective study in which the intraoperative force transmitted to the UPJ or ureter is directly captured would be the next step towards validating our conclusions.

Despite these limitations, this study demonstrates that postoperative ureteral obstruction after PCNL may be able to be predicted using simple objective variables. These results can be used to help guide tube management: patients who are at high risk for ureteral obstruction after PCNL may benefit from an indwelling ureteral stent to avoid a prolonged course with NT; conversely, patients who are at low risk for ureteral obstruction might be good candidates for totally tubeless PCNL.

## Conclusion

Ureteral obstruction after PCNL is associated with stone location, choice of access, and concurrent ipsilateral URS. Recognizing these risk factors can help determine which patients may benefit from postoperative renal drainage or which patients may be good candidates for totally tubeless PCNL.

## Supplementary Information

Below is the link to the electronic supplementary material.

Supplementary file 1 (DOCX 717 KB)

Supplementary file 2 (DOCX 417 KB)

Supplementary file 3 (DOCX 74 KB)
