# Supplementary material for: Determinants of ureteral obstruction after percutaneous nephrolithotomy
Source: Urolithiasis. 2022 Oct 14;50(6):759–64. doi: 10.1007/s00240-022-01365-8 (PMC9584844; doi:10.1007/s00240-022-01365-8)
Supplement: Supplementary file 1 — (DOCX 717 KB) [file 240_2022_1365_MOESM1_ESM.docx]

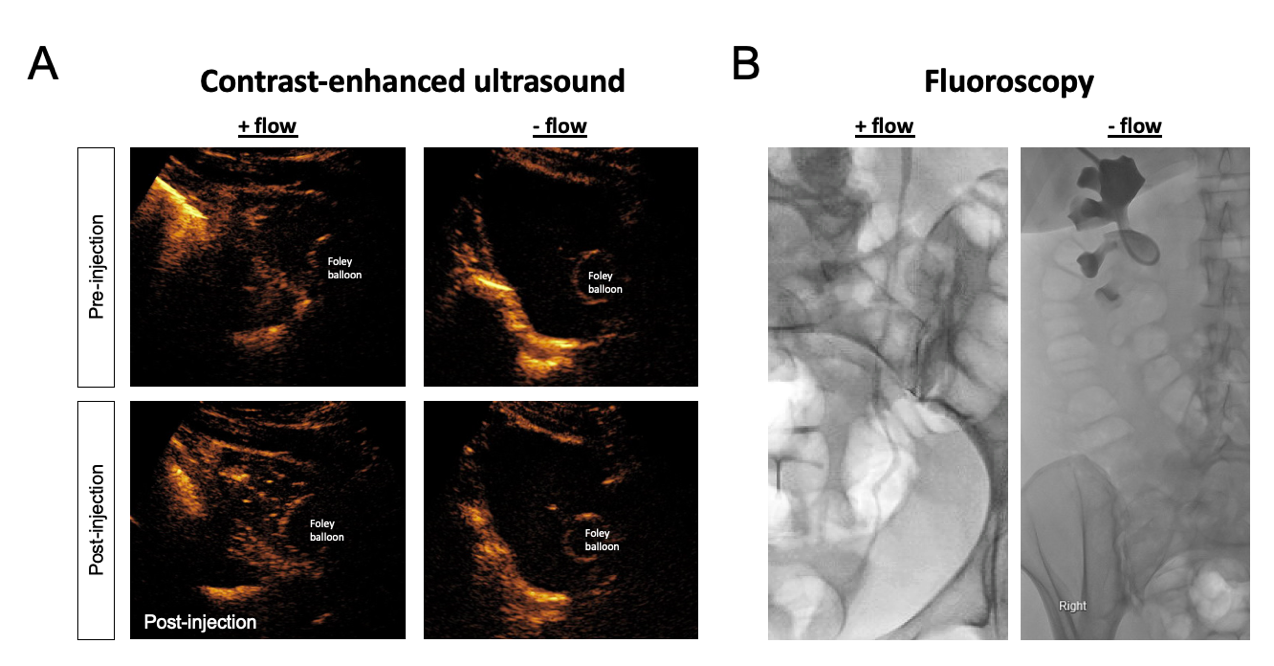


Supplemental Figure 1. Examples of positive and negative imaging results indicating the presence of flow.
