# Supplementary material for: Determinants of ureteral obstruction after percutaneous nephrolithotomy
Source: Urolithiasis. 2022 Oct 14;50(6):759–64. doi: 10.1007/s00240-022-01365-8 (PMC9584844; doi:10.1007/s00240-022-01365-8)
Supplement: Supplementary file 2 — (DOCX 417 KB) [file 240_2022_1365_MOESM2_ESM.docx]

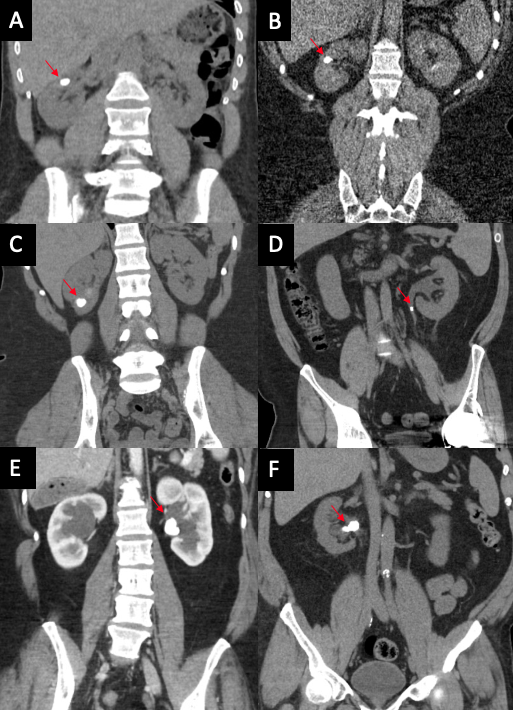


Supplemental Figure 2. Different stone locations indicated by red arrows. A) Upper pole stone. B) Mid pole stone. C) Lower pole stone. D) Ureteral stone. E & F) Renal pelvis stone.
