# Supplementary material for: Determinants of ureteral obstruction after percutaneous nephrolithotomy
Source: Urolithiasis. 2022 Oct 14;50(6):759–64. doi: 10.1007/s00240-022-01365-8 (PMC9584844; doi:10.1007/s00240-022-01365-8)
Supplement: Supplementary file 3 — (DOCX 74 KB) [file 240_2022_1365_MOESM3_ESM.docx]

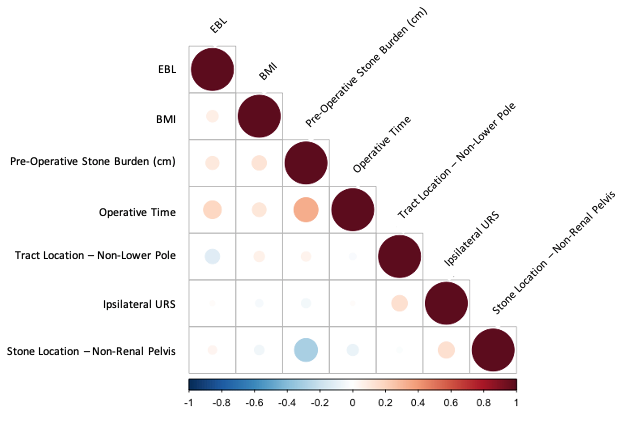


| **Correlation Coefficients** |  |  |  |  |  |  |  |
| --- | --- | --- | --- | --- | --- | --- | --- |
|  | EBL | BMI | Pre-Operative Stone Burden (cm) | Operative Time | Tract Location - Non-Lower Pole | Ipsilateral URS | Stone Location - Non-Renal Pelvis |
| EBL | 1 |  |  |  |  |  |  |
| BMI | 0.077 | 1 |  |  |  |  |  |
| Pre-Operative Stone Burden (cm) | 0.1 | 0.12 | 1 |  |  |  |  |
| Operative Time | 0.18 | 0.11 | 0.33 | 1 |  |  |  |
| Tract Location - Non-Lower Pole | -0.12 | 0.064 | 0.052 | -0.027 | 1 |  |  |
| Ipsilateral URS | 0.015 | -0.033 | -0.05 | 0.012 | 0.14 | 1 |  |
| Stone Location - Non-Renal Pelvis | 0.043 | -0.054 | -0.3 | -0.074 | -0.018 | 0.15 | 1 |
|  |  |  |  |  |  |  |  |
|  |  |  |  |  |  |  |  |
| **p-values** |  |  |  |  |  |  |  |
|  | EBL | BMI | Pre-Operative Stone Burden (cm) | Operative Time | Tract Location - Non-Lower Pole | Ipsilateral URS | Stone Location - Non-Renal Pelvis |
| EBL | 0 |  |  |  |  |  |  |
| BMI | 0.52 | 0 |  |  |  |  |  |
| Pre-Operative Stone Burden (cm) | 0.12 | 0.023 | 0 |  |  |  |  |
| Operative Time | 0.015 | 0.13 | 0.0000033 | 0 |  |  |  |
| Tract Location - Non-Lower Pole | 0.015 | 0.45 | 0.53 | 0.73 | 0 |  |  |
| Ipsilateral URS | 0.77 | 0.78 | 0.87 | 0.88 | 0.034 | 0 |  |
| Stone Location - Non-Renal Pelvis | 0.9 | 0.26 | 0.000053 | 0.32 | 0.57 | 0.015 | 0 |

Supplemental Figure 3. Correlation matrix graph and tables
